# Comparison of Modules of Wild Type and Mutant Huntingtin and TP53 Protein Interaction Networks: Implications in Biological Processes and Functions

**DOI:** 10.1371/journal.pone.0064838

**Published:** 2013-05-31

**Authors:** Mahashweta Basu, Nitai P. Bhattacharyya, Pradeep K. Mohanty

**Affiliations:** 1 Theoretical Condensed Matter Physics Division, Saha Institute of Nuclear Physics, Bidhan Nagar, Kolkata, India; 2 Crystallography and Molecular Biology Division, Saha Institute of Nuclear Physics, Bidhan Nagar, Kolkata, India; University of Zurich, Switzerland

## Abstract

Disease-causing mutations usually change the interacting partners of mutant proteins. In this article, we propose that the biological consequences of mutation are directly related to the alteration of corresponding protein protein interaction networks (PPIN). Mutation of Huntingtin (HTT) which causes Huntington's disease (HD) and mutations to TP53 which is associated with different cancers are studied as two example cases. We construct the PPIN of wild type and mutant proteins separately and identify the structural modules of each of the networks. The functional role of these modules are then assessed by Gene Ontology (GO) enrichment analysis for biological processes (BPs). We find that a large number of significantly enriched (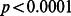) GO terms in mutant PPIN were absent in the wild type PPIN indicating the gain of BPs due to mutation. Similarly some of the GO terms enriched in wild type PPIN cease to exist in the modules of mutant PPIN, representing the loss. GO terms common in modules of mutant and wild type networks indicate both loss and gain of BPs. We further assign relevant biological function(s) to each module by classifying the enriched GO terms associated with it. It turns out that most of these biological functions in HTT networks are already known to be altered in HD and those of TP53 networks are altered in cancers. We argue that gain of BPs, and the corresponding biological functions, are due to new interacting partners acquired by mutant proteins. The methodology we adopt here could be applied to genetic diseases where mutations alter the ability of the protein to interact with other proteins.

## Introduction

Cellular functions are carried out by proteins interacting with other proteins and macromolecules like DNA, RNA, etc. It is believed [1] that the modular organization of cellular functions are related to the underlying modular structure of the protein protein interaction network (PPIN). Understanding PPIN would elucidate how such interactions execute basic functions in cells and may explain the abnormalities arising from mutations in genes. In particular, mutation at the binding site of a protein may lead to loss of it's ability to function together with existing interacting partner(s). On the other hand, mutation may also create regions where new protein partners can bind. Therefore, loss or gain of interaction due to mutation may contribute to causation, progression or modulation of disease. It has been reported recently [2] that out of 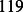 mutations in 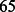 distinct diseases, 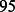 mutations result in loss of function (LOF), 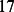 mutations result in gain of function (GOF) and 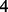 mutations changes the preferences for interaction. Based on this experimentally validated data, it has been predicted that 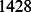 mutations might be related to interaction defect. Using the structural information at atomic levels either through crystallography or homology modeling, it has been shown that 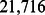 mutations in 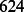 genes either alter amino acid sequences or produce truncated proteins. Among 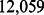 mutations that alter amino acid sequences, 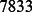 mutations are located in the interface of interaction with other proteins. Such mutations at interfaces of interactions may disrupt or enhance the interactions with the partners. This study also emphasizes the role of loss or gain of interactions of mutant proteins in human diseases. However, for such analysis, it is necessary to have structural information at atomic levels, which may be achieved if 3-dimensional structures of the proteins or their homologs are known. But, for the most of the protein protein interactions such information is not available [3]. Moreover, very little is known about the role of such altered interactions in corresponding pathological conditions. It remains a challenge to relate genetic mutation data to PPIN and to understand molecular cause of disease. In the present communication, we probe whether gain or loss of interactions of mutant Huntingtin protein (HTT) that causes Huntington's disease (HD) can explain functional abnormalities observed in HD. We have also used the same approach to find how loss or gain of interactions of mutant TP53 in cancers may result in alterations of functions.

## Analysis and Results

### Mutation in HTT Protein

Huntington's Disease (OMIM ID: 143100) is a rare autosomal dominant progressive degenerative neurological disease caused by expansion of normally polymorphic CAG repeats beyond 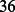 at the exon1 of the gene Huntingtin (HTT) [4]. Over the years, various cellular processes/conditions like excitotoxicity, oxidative stress, mitochondrial dysfunction, endoplasmic reticulum stress, axonal transport, ubiquitin proteasome system, autophagy, transcriptional deregulation and apoptosis have been implicated in HD pathology [5], [6]. Even though GOF was inferred initially from the autosomal dominant nature of transmittance of the disease, the underlying molecular details still remain largely unknown. Inverse correlations between age at onset and number of CAG repeat beyond 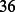 in HTT gene, increased aggregates of mutant HTT (mHTT) and apoptosis, correlation of CAG repeat numbers in HTT gene with levels of ATP/ADP and altered expression of few genes [4], [7]–[10] suggest toxic GOF of mutant protein that disrupts normal cellular functions and causes neuronal death. Mutant HTT preferentially interacts with DNA sequences, alters conformation of DNA facilitating binding of other transcription factors to the specific sequences and modulates transcription of genes. This result also indicates a dominant GOF of mHTT [11]. Wild type HTT (wHTT) is known to be involved in protection of apoptosis [12]–[15], regulation of gene expression [16], [17], mitosis and neurogenesis [18], neuronal development [19] and maintenance of body weight [20]; all these processes are altered in HD [5], [6]. These results indicate that loss of one of the alleles in HD could contribute to increased apoptosis and altered gene expressions observed in HD. LOF of wild type protein may thus contribute, at least partially, to HD pathology [21]. There are also several experimental evidences available against simple LOF(s) of wild type HTT [22]–[25].

#### Construction of HTT-interacting protein network

We have collected the HTT interacting proteins from published data and find that 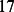 proteins preferentially interact with wHTT, while 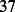 proteins are either identified in aggregates of mHTT only or interact preferentially with mHTT (the references for each of the observations are provided in Dataset S1 (sheet 1) and in Text S1 (Text 1)). These 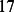 and 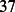 proteins are referred to as the primary interactors of wHTT and mHTT respectively. Next, we assimilate interacting partners of these primary interactors from BioGrid (Version 3.1.88, May 2012), a public database that contains genetic and protein protein interaction data for humans and other organisms [26]. In the present study, we have considered both physical and genetic interactions (refer to the section 'Robustness analysis' for details). It turns out that there are 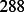 secondary interactors of wHTT (proteins which interact with the 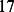 primary interactors), whereas there are 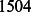 secondary proteins which interact with 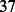 primary interactors of mHTT. The PPIN of wHTT interacting proteins is then constructed by considering all these 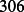 proteins (wHTT+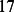 primary+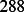 secondary interactors of wHTT) as nodes of the network; two nodes are connected if corresponding pair of proteins are found to be interacting partners of each other in BioGrid. Altogether there are 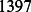 interactions in wHTT network which are listed in Dataset S1 (sheet 2). Similarly the PPIN of mHTT is constructed with 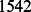 nodes (mHTT+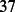 primary+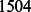 secondary interactors of mHTT) which has 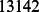 interactions from BioGrid (Dataset S1 (sheet 3)). We have used Cytoscape [27] for visual presentation of the wHTT and mHTT networks, which are shown in Fig. S1 in Text S1. Both the networks are densely interconnected and the nodes are too tangled there to find any apparent or obvious modular structures.

#### Characteristics of networks

A quantifiable description of these networks can be obtained by using graph theory, which provides several measures for comparison and characterization of complex networks. The most elementary characteristic of a node is its degree, 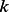, which represents the number of other nodes (proteins) it is connected with. The degree distribution, 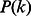, gives the probability that a randomly selected node has exactly 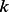 links. We find that both the wild and mutant PPINs follow a power law degree distribution, 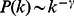 (Fig. S3 in Text S1) with exponents 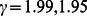 and average degrees 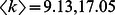 respectively. Another important quantity is the clustering coefficient which characterizes how connected are the neighbors of a given node. It is observed that the average clustering coefficient 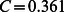 for mHTT network is lower compared to 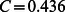 for wHTT PPIN. This indicates that, the former network is less compact and the interacting partners of the proteins are poorly connected among themselves. We have also calculated the average shortest path length 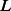, and the network diameter 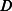 (listed in Table S2 in Text S1), which describe the structural properties of the network. The detailed definitions of 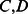 and 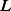 along with their evaluation procedure is illustrated in Text S1 (Text 2).

#### Gain and loss of interactions due to mutation

**Figure 1:**
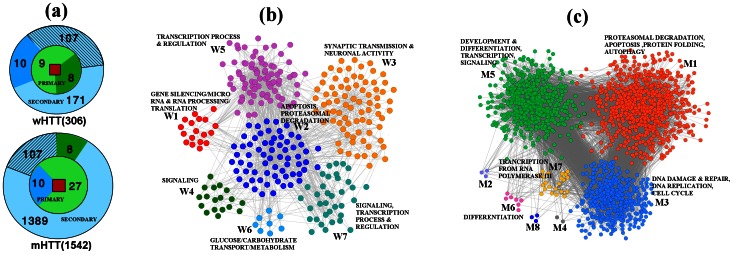
Construction and modularization of wild type and mutant HTT networks. (a) Proteins involved in the wHTT and mHTT networks: wHTT (mHTT) protein (red square) has 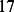 (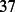) primary and 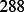 (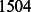) secondary interactors, shown schematically as the inner and outer circles. Of the 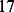 primary interactors of wHTT, 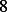 proteins (deep green) become secondary interactors of mHTT. Among the 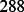 secondary interactors of wHTT, 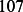 (shaded) proteins remain as the secondary interactor of mHTT whereas 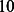 proteins (deep blue) becomes the primary interactors of mHTT. (b) and (c) Modules of the wHTT and mHTT networks from NGM algorithm, which yields 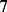 (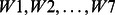) and 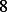 modules (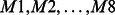) respectively are shown along with the relevant biological functions (obtained GO term enrichment analysis from GeneCodis3). Significant functions associated with the modules are also shown. Details of the GO terms are shown in Table S2 and Table S3 of the Supporting Text, respectively for wHTT and mHTT.

A closer look at PPINs of wHTT and mHTT reveals that among the 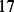 primary interactors of wHTT, 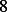 proteins still appear in PPIN of mHTT as secondary interactors, 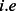. they interact with some of the primary interactors of mHTT. Again, among 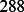 secondary interactors of wHTT, 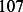 proteins are secondary interactors of mHTT, 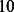 proteins interact directly with mHTT and the rest 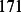 proteins do not take part in PPIN of mHTT (see Fig. 1(a)). Evidently, the mutant HTT network has gained several new interactions, 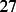 proteins as primary interactors and 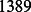 proteins as secondary interactors. This result is shown schematically in Fig. 1(a) and the detailed list of these proteins is given in Text 1 and Table S1 in Text S1. Since mutation of HTT has changed the PPIN substantially one expects a significant change in its functions.

#### Modules of wHTT and mHTT networks

There are several methods for obtaining natural modules of a network (or partitions of a graph) [28]. We adopt Newman-Girvan's modularization (NGM) algorithm [29], a commonly used method, to detect the modules of wHTT and mHTT networks. This algorithm partitions the network in a way that the intra-module connections between nodes are maximized in comparison to the inter-module connections. To find the modules, Newman and Girvan [30] proposed a score called modularity 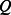 for every possible partition of a network; the maximum value of 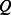 corresponds to the best partition. The details of the NGM algorithm for maximization of 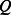 is described in Text S1 (Text 2). The NGM algorithm modularizes the PPIN of wHTT into 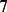 modules of sizes (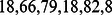 and 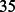) (see Table S2 in Text S1), with modularity 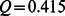, whereas PPIN of mHTT is partitioned into 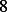 modules of sizes (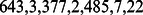 and 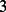) with 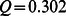. Modules of wHTT and mHTT networks are denoted by 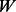 and 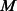 respectively. Figures 1(b) and (c) represent the modularized networks; all proteins belonging to a given module are shown in same color. Clearly, the mHTT network is visibly more complex than that of wHTT, which is consistent with the fact that it has a lower 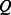 value [31].

#### Similarity between the modules

**Figure 2:**
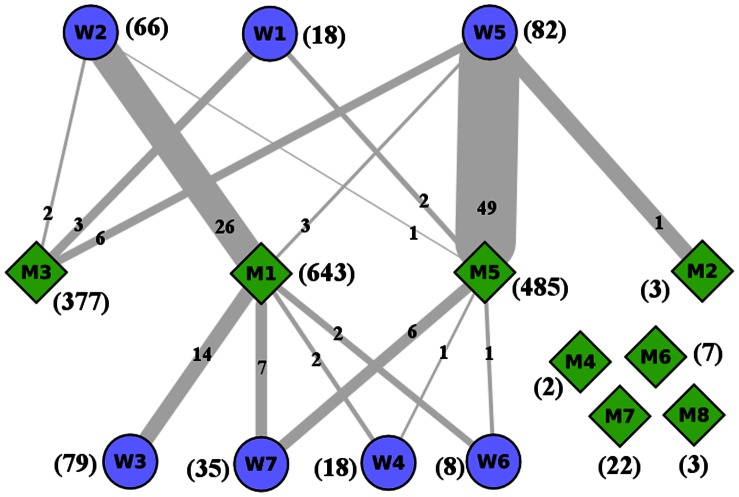
Similarity between modules of wHTT and mHTT networks. The figure describes pictorially the closeness between the modules of wHTT and mHTT PPIN; the modules having common protein or common GO terms are joined with edges (numerical value written on the edge as: common proteins).

Once the wild type and mutant networks are modularized, it is important to ask how similar is a module of wild type network with that of mutant network, in terms of their protein constituents. Mutant and wild type HTT networks have 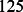 proteins common between them. After both the networks are modularized, these common proteins are distributed among the pair of wHTT- mHTT modules. For example, the module 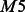 (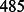 proteins) has 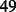 proteins in common with 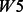 (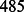 proteins), whereas it has only one common protein in 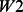 (out of 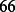 proteins) and two common proteins in 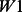 (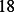 proteins). The detailed distribution of common proteins among wild and mutant modules of HTT are shown in Fig. 2.

To calculate the similarity among modules, first we construct a unique set of proteins from combining the proteins involved in the wild and mutant networks. This set consists of 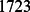 proteins in case of HTT. Now every module of wHTT and mHTT are considered as a unique 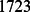 dimensional vector as follows. Each protein is identified with a specific position in the vector; presence (or absence) of a specific protein in a module, say 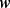, is mapped on to a corresponding vector 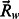 by inserting 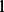 (or 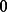) at respective position. A similarity measure between a pair of modules 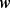 and 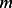 is well represented by the angle 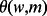 between the corresponding vectors 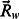 and 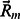,
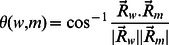



It is rather simpler to use 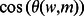 as the similarity measure as 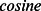 function is monotonic in the range 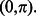 It is easy to see that if the modules have 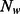 and 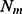 proteins individually and 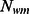 protein in common, the similarity measure is
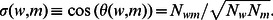
(1)


Clearly 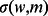 varies in the range 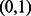 with maximum value 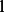 corresponding to the fact that the modules are identical, 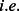 they have same set of proteins.

In Fig. 2, we represent the similarity among modules of mHTT and wHTT as a bipartite network with links having thickness proportional to 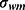. The thickest link between 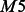 and 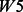 indicates that these modules are significantly similar. For examples, the module 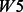 has 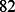 proteins and 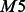 has 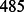 proteins; 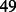 proteins are common among the proteins in these 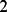 modules; thus the protein similarity index for 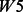-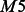 pair is 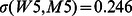. Similarly among 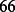 proteins in 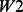 and 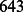 proteins in 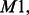 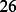 proteins are common (corresponding 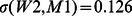).

#### Enrichment of GO terms for biological process

It has been observed that the proteins identified in a particular complex are involved in similar functions [32]. From network perspective, these complexes are represented by modules and they appear as distinct group of nodes which are highly interconnected with each other but have only a few connections with the nodes outside of the module. It is important to ask, if such a structural partition relates to any functional enrichment. Among many bioinformatics tools available for such analysis [34] we utilize GeneCodis3 [33] (explained in Text S1 (Text 3)) to obtain the possible Biological processes enriched by the proteins in a given module. Given a query set of proteins GeneCodis3 provides the enriched biological process, molecular functions, and cellular components as defined by the Gene ontology. Biological process in Gene ontology is described as a series of events carried out by one or more ordered assemblies of molecular functions [35]. The proteins in each module are used as input to GeneCodis3 [33] and significantly enriched GO terms for BPs obtained using 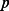-values calculated through Hypergeometric analysis corrected for false discovery rate (FDR). Results of enrichment analyses for 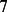 modules of wHTT and 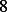 modules of mHTT network are shown in Datasets S2 and S3 respectively.

Since many proteins are known to be involved in a particular BP, and a given protein may also contribute to multiple BPs, it is likely that proteins in different modules in wHTT and mHTT network participate in a specific BP due to either overlap in proteins or BPs. To identify the overlaps of BPs between modules in wHTT and mHTT networks, we separately identify the common GO terms between the wHTT and the mHTT modules. It is evident from Datasets S4 (sheet 2) that 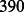 unique GO terms are being enriched (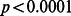) due to proteins in modules of mHTT network, while 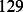 GO terms are enriched with proteins in the modules of wHTT network (Datasets S2 (sheet 1)). Among the GO terms present in wHTT and mHTT network, 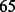 are common. As a result due to mutation, 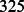 GO terms are gained by mHTT and 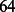 GO terms are lost by wHTT. The common 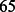 GO terms represents both gain and loss.

For convenience, we clubbed the the GO terms in a given module to broadly assign one or more appropriate biological function(s). For example, GO:0010506 (regulation of autophagy), GO:0016559 (peroxisome fission), GO:0031929 (TOR signaling cascade), GO:0000045 (autophagic vacuole assembly), GO:0006897 (endocytosis) in module 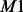 are bought under a single biological function ''Autophagy''. Similarly in module 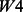 GO:0043507 (positive regulation of JUN kinase activity), GO:0072383 (plus-end-directed vesicle transport along microtubule), GO:0046330 (positive regulation of JNK cascade), GO:0046328 (regulation of JNK cascade) are clubbed under ''Signaling''. The assigned biological functions for modules of wHTT and mHTT are shown in Fig. 1(b) and (c) (details are given in Datasets S4).

#### Gain and loss of biological process in HTT networks

Comparison of enriched BPs in the modules of wHTT and mHTT reveal that the mHTT network has acquired several new BPs which were absent in wHTT, indicating gain of biological processes. Similarly enriched BPs of wHTT which are not present in mHTT are lost. Hence biological functions carried out by the BPs which are gained or lost in mHTT networks may result in functional gain or loss due to mutation in HTT.

*Gain of biological process:* The unique GO terms enriched in the modules of mHTT networks are listed in Datasets S4 (sheet 2) and in Text S1 (Table S3). The GO terms in module 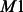 are related to cell cycle (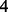 GO terms), signaling (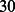), transcription processes and regulation (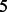), apoptosis (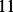), DNA damage and repair (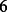), Immunological (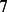), protein folding (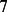), autophagy (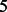), translation (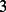), metabolism (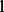), development and differentiation (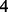), cell migration and shape (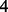), proteasomal degradation (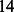), Protein complex/membrane assembly/stabilization (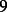) and others (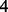). It is known that many of these processes are involved in HD pathogenesis [36]. In 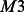, the enriched GO terms are assigned to DNA repair (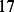), Transcription processes and regulation (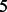), DNA replication (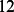), cell cycle (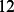) and others (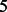). Note that, it has been shown recently that DNA repair, replication and cell cycle are involved in HD. In fact, activation of DNA synthesis and cell cycle increase apoptosis in terminally differentiated neuronal cells, instead of increasing cell division [37], [38]. Besides, recent studies have explored the role of DNA repair in neurodegenerative disease [39] and show that interaction of mHTT with Ku70/XRCC6 impairs repair activity [40]. A large number of GO terms related to development and differentiation (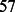 GO term), transcription process and regulation (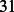), cell cycle (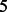), DNA damage and repair (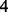), Carbohydrate/Glucose transport/metabolism (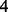), Cell growth (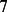), signaling (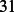) and others (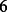) are enriched in module 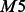. The role of development and differentiation in HD is not clear. However recent studies in HD [19], [41] indicate that neurogenesis is possibly altered and differentiation/development could be defective. Deregulation of transcription is considered to be one of the most important abnormalities in HD [42]. GO terms related to differentiation are also enriched with proteins in module 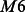, although the terms are distinct from that in module 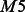. All 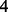 GO terms enriched in 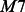 are related to transcription by RNA polymerase III. It is known that both tRNA and some miRNAs [43] are synthesized by RNA polymerase III, however their role in HD is unknown. Thus it is evident that the the protein interactions gained in mHTT network result in enrichment of the biological processes in its modules.

*Loss of biological process:* The unique GO terms enriched in the modules of wHTT which are absent in the modules of mHTT network represent the loss of functions due to mutation in HTT protein. The 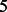 GO terms in 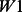 include gene silencing, micro RNA processing and translational regulation. The GO terms relating to proteasomal degradation (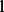 GO term), cell cycle (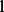), apoptosis (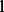) and circadian rhythm (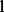) are present in 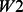. Similarly, signaling (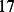 GO terms), synaptic transmission, neuronal activities (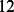) transport (ion/sugar) (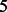) and others (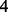) are associated with module 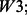 glucose/carbohydrate transport and metabolism (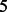), cell cycle (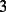) and protein/transmembrane transport (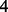) with 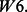 In 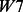 only one GO term describing transcription processes and regulation is enriched. The GO terms and the associated BPs that are lost due to mutation are provided in Datasets S4 (sheet 1) and in Text S1 (Table S3) respectively.

We have clubbed the relevant GO terms to represent signaling, transcription process and regulation, apoptosis, cell cycle etc. (refer to Table S3 of Text S1). For example, GO terms (GO:0000088) and (GO:0000236, GO:0000087, GO:0007091) which are enriched in 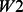 and 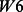 respectively relates to cell cycle. Similarly the 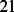 GO terms which are enriched in 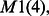 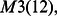 and 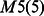 (Datasets S4 (sheet 2)) are also associated to cell cycle. Although cell cycle is enriched in both wHTT and mHTT modules, no GO terms are common among them. Thus, the loss of interaction with wHTT may result in loss of above 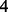 GO terms in wild type network resulting in LOF, whereas the gain of interaction with mHTT may be associated with gain of these 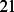 GO terms relating to GOF of cell cycle.

It is interesting to note (from Table S3 of Text S1) that the GO terms related to DNA replication, protein folding, autophagy, cell growth are only observed in the modules of mHTT networks. So these processes are gained due to new interaction with mHTT. Similarly, GO terms related to gene silencing/microRNA processing/translation, transport (ion/protein/sugar etc) are observed in wHTT network only. Therefore, loss of interaction with wHTT may result in the loss of these BPs in HD.

*Both loss and gain of biological process:* Modules in wHTT and mHTT networks have several proteins or GO terms common among them, which indicate loss as well as gain of functions and support the notion that both loss and gain may occur due to mutation in HTT [21]. For example, modules (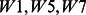) and (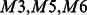) have 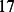 enriched GO terms related to transcription processes and regulation. Similarly, modules (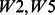) and (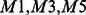) share 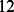 enriched common GO terms related to apoptosis and 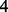 common GO terms relating to cell cycle. Thus, the general function of transcription and apoptosis could arise from loss as well as gain of interactions of mHTT protein. The details of the functions associated with the 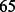 GO terms (common between wHTT and mHTT) are presented in Datasets S4 (sheet 3) and Table S3 of Text S1, they correspond to the gain and loss of functions in the HD.

From the above analysis we observe that most of the functions that are enriched in the modules of wHTT and mHTT networks are altered in the pathogenesis of HD. The post transcriptional regulation of genes, associated with module 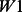 of wHTT network, can be related to negative regulation of gene expression by the non-coding RNAs like micro RNAs, which are well documented [44]. Role of apoptosis [5], [36], synaptic transmission [45], JNK pathway [46], transcription deregulation [42], glucose transport [47], [48], estrogen [49] and various types of epigenetic changes including histone modifications in different neurological diseases [50] in HD pathogenesis have also been reported.

In summary, many new BPs (GO terms) appear in the mHTT network and some of the BPs present in wHTT network are lost; a few are found to be common between modules of wHTT and mHTT. As a result some biological functions involving the enriched GO terms are gained by mHTT and a few are lost from the modules of wHTT. This provides molecular mechanism of the gain and/or loss of functions observed in HD pathogenesis.

### Mutation in TP53 Protein

TP53 protein, initially identified as an oncogene, is now established as a tumor suppressor gene which participates in diverse cellular functions like transcription regulation, DNA repair, apoptosis, and genome stability, and many others. Mutation to TP53 is identified in more than 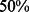 of the tumors. It is evident from COSMIC database [51] that R175H, R273H and R248W mutations of TP53 are the most prevalent ones. Since TP53 is a tumor suppressor gene, it is expected that its mutations might result in the LOF of the wild type protein. Some mutations of TP53 are also known to attain new function(s) [52], [53]. For example, exogenous expression of mutant TP53 (R273H and others) in mouse cells devoid of endogenous TP53 results in several cellular phenotypes of cancers [54]–[56]. To understand the underlying molecular mechanism of GOF of mutant TP53, it was recently shown [57] that nardilysin (NRD1) protein, which does not interact with wild type TP53 but interacts only with mutant TP53 (R273H), may contribute to the metastatic properties of this mutant protein.

#### PPIN of wTP53 and R273H mutant TP53 (mTP53)

In a recent study [57], it has been shown that 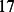 proteins preferentially interact with the wild type TP53 (wTP53) and 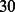 other proteins interact exclusively with mutant TP53 (mTP53). To construct the protein interaction networks we take these primary interacting proteins of wTP53 and mTP53 and consider their interacting partners existing in BioGrid database [26]. The detailed protein interaction data are given in the Datasets S5. The PPIN is constructed separately for wTP53 and mTP53, as described for HTT. It turns out that wTP53 has 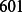 secondary interactors whereas mTP53 has only 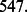 Thus the PPIN of wTP53 and mTP53 are constructed taking 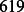 proteins 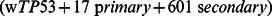 and 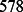 proteins 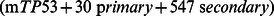 respectively. Both the networks (shown in Fig. S2 in Text S1) are found to be densely packed with similar structural properties. Their degree distributions are scale free (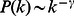) with the exponents 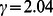 (wTP53) and 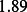 (mTP53) (Fig. S3 in Text S1) and average degree 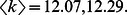 The other network properties, like the average clustering coefficient 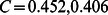, the diameter of the networks 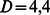 are also comparable (listed in Table S2 in Text S1).

**Figure 3:**
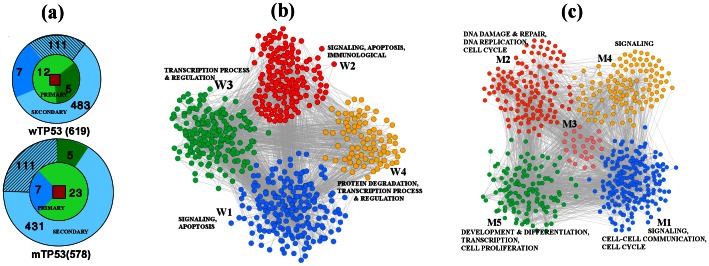
Construction and modularization of wild type and mutant TP53 networks. (a) Proteins in wTP53 and mTP53 networks: wTP53 (mTP53) protein (red square) has 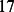 (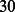) primary and 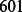 (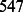) secondary interactors, represented by the inner and outer circles. Only 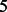 (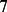) primary (secondary) proteins of wTP53 interact with mTP53 as secondary (primary) interactors. Again 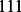 secondary proteins of wTP53 remain as secondary interactors of mTP53. (b) and (c) shows the modules of wTP53 and mTP53 network along with few plausible candidate BPs. Details of the GO terms are shown for wTP53 and mTP53 respectively in Table S6 and Table S7 of Text S1.

The change in interactions and the interacting partners due to mutation of TP53 is shown schematically in Fig. 3(a). Of 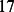 primary interactors of wTP53, only 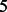 proteins remain involved in mutant network as secondary interactors of mTP53 and the remaining 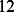 do not interact with mTP53. Among the 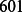 secondary interactors of wTP53, 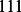 proteins remain as a secondary interactor of mTP53 and 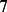 of them interact directly, 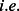 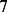 secondary interactors of wTP53 become primary interactors of mTP53. Lists of these proteins are given in Text 1 and Table S1 in Text S1.

#### Modules of wTP53 and mTP53 networks

**Figure 4:**
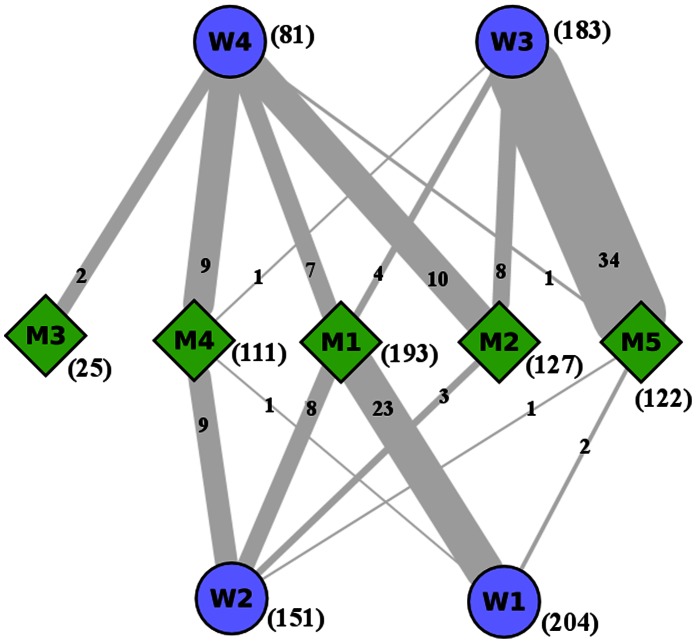
Similarity between modules of wTP53 and mTP53 networks. The bipartite network constructed with the modules of wTP53 and mTP53; the common proteins present between a pair of wild and mutant module is written on respective link. The number of proteins that constitute the modules are written beside it.

In order to identify the modules of the wTP53 and mTP53 networks, we use NGM algorithm [29]. It turns out that PPIN of wTP53 is modularized into 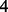 modules of size 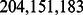 and 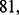 whereas mTP53 network gives 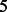 modules of size 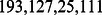 and 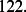 The corresponding modularity values are 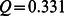 and 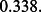 Figures 3(b) and (c) show the modules of wTP53 and mTP53 with different colours. Each module of wTP53 or mTP53 has unique set of protein. However, there is a large overlap of secondary interactors (proteins which do not interact directly with TP53) in the wTP53 and mTP53 networks, which is distributed among different modules (in total 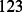). We observe that among 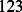 common proteins, 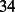 belong to module 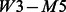, whereas module pairs 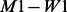 (and 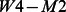) have 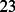 (and 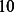) common proteins. One can define a similarity measure 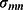 using Eq. (1) for every pair of wTP53-mTP53 modules. Taking the similarity indices 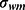 as weights (or thickness) of the link we have constructed a bipartite network which is shown in Fig. 4; the number of proteins is written beside each of the modules and the number of common proteins is specified along the links.

Enrichment of biological processes for the proteins present in every module of wTP53 and mTP53 PPIN using GeneCodis3 are presented in Datasets S6 and S7 respectively, where only the GO terms with 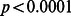 are considered. The number of enriched GO terms in modules of wTP53 PPIN are 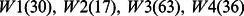 and those for mTP53 are 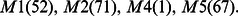 Note that module 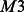 has no GO terms enriched with 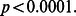


#### Loss and gain of biological processes in TP53 networks

Enrichment analysis of proteins in modules of wTP53 and mTP53 using GeneCodis3 reveals that respectively 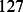 and 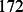 GO terms (or biological processes) are enriched significantly 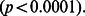 Among 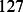 GO terms of wTP53 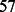 GO terms do not appear in the mTP53 representing loss of the corresponding biological processes. Again the mTP53 network has 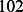 new GO terms (which were absent in wTP53). Besides, 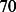 enriched GO terms are found to be common in modules of mTP53 and wTP53 networks. We further associate each of the enriched GO terms with a relevent function. Loss and gain of these broadly classified functions are discussed below.

*Gain of biological processes:* The biological processes related to 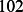 new GO terms of mTP53 are gained due to mutation. The functions enriched in module 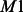 of mTP53 network are cell-cell communication (no of GO terms 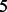), signaling (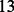), protein complex/membrane assembly/stabilization (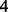), proteasomal degradation (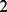), cell cycle (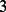), DNA damage and repair (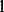) and others (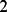). GO terms related to DNA replication (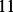), DNA damage and repair (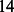), cell cycle (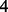), immunological functions (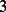), proteasomal degradation (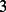) and signaling (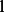) are enriched in 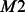. Similarly GO terms related to differentiation and development (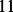), signaling (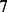), transcription (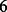), cell proliferation (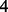), apoptosis (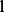), cell cycle (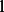) and DNA damage (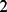) and others (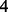) are enriched with proteins in module 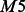. The extensive list of the GOF is given in Dataset S8 (sheet 2) and in Table S4 in Text S1. Thus new functions carried out by these biological processes are due to gain of interaction.

*Loss of biological processes:* On the other hand some of the enriched GO terms of wTP53 are absent in the mutant network. Corresponding biological processes are lost due to mutation in TP53. Altogether 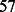 unique GO terms are enriched with proteins in modules of wTP53 networks which are classified into broad class of functions (see Dataset S8 (sheet 1) and Table S4 in Text S1. The resulting loss of biological functions in various modules are, 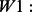 signaling (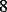), proteasomal degradation (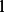), translation (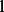), cell migration and movement (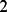) and others (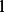); 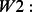 signaling (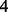), apoptosis (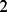) and immunological (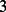); 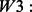 cell cycle (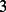), signaling (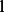), transcription process and regulation (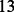), DNA replication (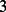), DNA damage and repair (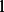); 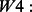 transcription process and regulation (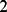), proteasomal degradation (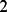), translation (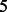) and others (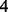); 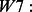 transcription process and regulation (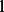).

*Both loss and gain of biological processes:* The 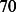 GO terms common between wTP53 and mTP53 networks are related to the functions, cell cycle (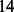 GO terms), transcription (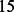), DNA damage and repair (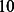), cell growth (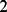) and apoptosis (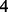), signaling (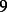), DNA replication (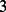), proteasomal degradation (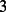), immunological (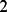), development and differentiation (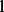), metabolism (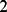) and others (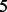). Thus these functions are possibly enriched due to both gain and loss of interactions (details are shown in Dataset S8 (sheet 3) and in Table S4 in Text S1.

#### Analysis of proteins in different modules using tool GeneDecks

Recently metastasis has been shown as the GOF as R273H cells attain metastatic property in cell model [57]. Since metastasis is not described as a “biological process” in Gene Ontology term, we have used another tool, GeneDecks [68], which provides a similarity metric by highlighting shared descriptors between genes, based on annotation within the GeneCards compendium of human genes (see Text 4 in Text S1 for details). Taking the proteins of the modules of wTP53 and mTP53 separately as a query field, we look for “metastasis” in the attribute “disorder” among many other descriptors which are enriched for different types of cancers (Dataset S9). It is observed that the descriptor “metastasis” is enriched with the protein modules 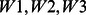 of wTP53 network and all the modules (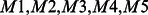) of mTP53 network. Thus, the loss of interactions of proteins in the modules 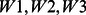 of wTP53 due to mutation may result in the LOFs related to metastasis. Similarly, the gain of interactions of proteins in all the modules of mTP53 may result in the GOFs related to metastasis.

That LOF of wTP53 and GOF of mTP53 may contribute to invasion and metastasis, is reviewed recently [69]. TP53 mutations at the DNA binding domain are common and such mutations suppress expression of target genes. It is supported by several experiments [69] that suppression of transcriptional program for genes involved in epithelial-mesenchymal transition (EMT) may contribute to induction of EMT resulting in metastasis. Further, it is ascertained that loss of functions in wTP53 lead to increased cell motility in various cell types, and increased expression of fibronectin, collagens and extracellular matrix (ECM) proteins. Enhanced expression of these proteins potentially increase the interaction between cells and ECM. LOF in wTP53 also activate Rho GTPases and modulates cell migration [69].

Role of mTP53 in metastasis has been established in many other studies. Mutant TP53 (R175H) is involved in TGF mediated invasion and metastasis in breast cancer cells through TP63 and SMAD3 [55]. Note that, in our analysis, SMAD is present in module 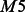 of mTP53 network. It is known that mutant TP53 (R175H and R273H) increases endocytic recycling of adhesion molecule integrin and EGFR promoting and metastasis [56], [70]. Mutation in TP53 also activate EGFR/PI3K/AKT pathways and thereby increases invasion [71]. Various other mechanisms of increased metastasis by the mutant TP53 have also been studied [69]. Thus the gain of biological processes obtained from the analysis of mTP53 protein networks provides an explanation of GOFs observed in cancers.

### Robustness Analysis

In general, the modularization methods partition the network into communities of proteins which are densely connected. Thus in a large network it is quite expected that deletion of small fraction of links, whether selected methodically or randomly, does not alter the overall structure significantly. In fact, the degree distributions of all four networks studied here (namely PPIN of wHTT, mHTT, wTP53 and mTP53) are scale free (see Fig. S3 of Text S1), and it is known that such scale free networks are robust against random removal of nodes or links, but they could be fragile against targeted attack [72].

Again, since several databases of protein interactions largely overlap [73] in their contents, it is natural to expect that the broadly classified biological functions obtained here for HTT and TP53 networks would not differ substantially. In this study we used Biogrid [26] for creating the differential PPIN of the wild type and mutant HTT and TP53 proteins by connecting every pair of proteins which are listed in BioGrid as interacting partner of each other. This includes experimentally validated genetic and physical interactions. To check the robustness of our analysis, first let us remove all genetic interactions listed in BioGrid. This reduces the total number of protein interactions of BioGrid to 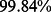, whereas the interactions of wHTT, mHTT, wTP53 and mTP53 are reduced to 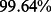, 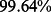, 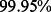 and 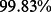 respectively (see Table 1). Among the other experiments considered in BioGrid, Yeast 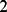 Hybrid (Y2H) assay results in larger false positives [74]. Thus we further remove all the interactions which are identified only once by Y2H. This stringent criterion consequently reduces both the number of interactions and the number of proteins by 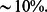 The total number of interactions of BioGrid is, however, reduced by 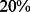. Since the wild type and mutant networks are altered only a little compared to the expected value 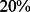, one expects that deletion of a small fraction of interactions will not change the network properties significantly.

**Table 1:** Change in the total number of proteins and the interactions after excluding (a) genetic interactions and then (b) excluding interactions which are validated by only one Y2H experiment.

	Total no. of interactions	(a)Excluding genetic(%)	(b)Excluding genetic & Y2H(%)	Total no. of proteins	Excluding genetic & Y2H(%)
**PPIN(human)**	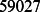	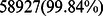	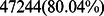	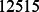	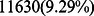
**wHTT**	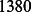	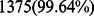	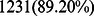	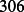	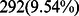
**mHTT**	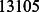	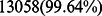	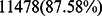	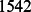	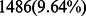
**wTP53**	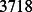	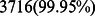	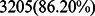	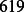	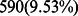
**mTP53**	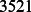	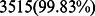	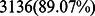	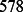	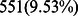

To demonstrate this explicitly, we reconstruct the PPIN of mHTT keeping only the reduced set of interactions and then identify the protein modules using Newman Girvan algorithm. The enriched GO terms (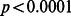) from GeneCodis3 shows that every module of mHTT (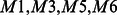 and 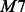) has significant protein overlap with only 'one distinct module' of the reduced network, which is referred to as the 'most similar module' (MSM) henceforth. The number of overlapping proteins and GO terms between the modules of mHTT and their corresponding MSM in the reduced network are listed in Table 2. Evidently, in all cases, about 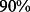 of the GO terms are retained. Thus, the loss, gain and loss/gain of biological processes obtained from BioGrid are quite robust.

**Table 2:** Comparison of number of proteins and GO terms in the modules of mHTT with respective of 'most similar module' of the network (a) after excluding genetic and Y2H experiments and (b) after deletion of 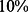 links.

	mHTT Module		(a) Excluding genetic & Y2H		(b) Random deletion of 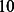 % links	
			MSM	Common ( 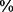 )	MSM	Common ( 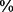 )
**M1**	No. of Proteins:	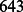	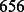	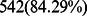	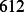	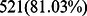
	No. of GO terms:	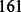	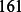	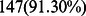	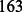	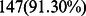
**M3**	No. of Proteins:	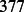	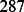	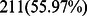	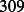	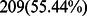
	No. of GO terms:	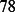	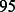	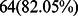	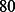	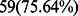
**M5**	No. of Proteins:	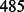	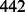	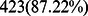	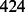	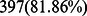
	No. of GO terms:	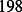	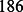	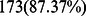	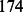	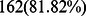
**M6**	No. of Proteins:	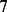	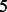	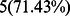	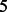	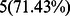
	No. of GO terms:	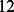	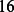	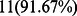	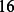	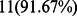
**M7**	No. of Proteins:	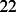	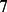	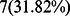	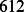	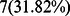
	No. of GO terms:	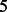	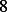	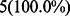	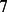	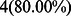

For completeness, we also removed randomly 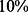 links of mHTT network and repeat the above analysis which is summarized in Table 2. Again, we find that about 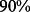 of the GO terms enriched in this network are identical to those obtained for mHTT. Thus, in general, the enriched biological processes obtained through this analysis are quite robust.

## Discussion and Conclusion

**Figure 5:**
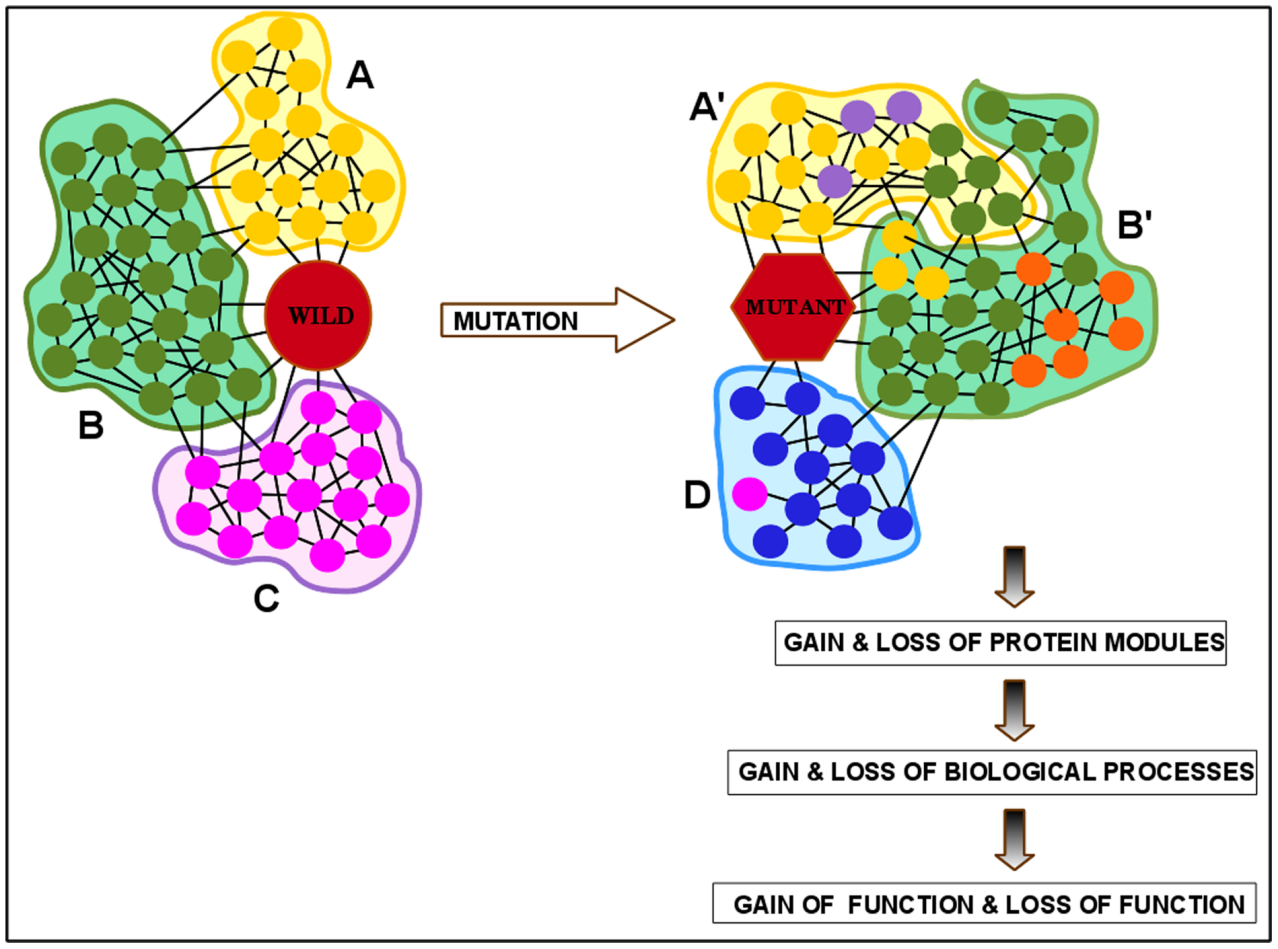
Loss and gain of functions from differential network studies. The general philosophy of the current work is described here for a schematic protein interaction network, where the wild type and the mutant protein have 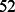 and 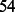 interactors respectively. There are three modules in wild type network (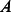, 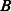, and 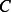); all proteins in a given module are marked with same colour. After mutation the protein looses some interactors (marked as pink) and gain some new ones (marked as orange, blue and violet). The PPIN of mutant protein has three modules 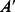, 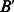 and 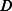. Module 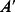, which primarily contains proteins of module 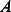, has some proteins from module 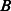 (green) and some new proteins (violet). Most of the proteins in module 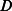 are new interactors and thus this module is gained by the mutation. Similarly proteins of module 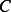 have lost their interactions. Correspondingly, the BPs which are enriched in module 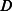 are gained and those enriched in module 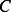 are lost. This loss or gain of BPs lead to loss or gain of functions in the pathogenesis of the mutation causing disease.

Mutation in protein may change its preference for binding with other proteins and alter the corresponding PPIN substantially. We use a graph theory based modularization approach to identify the modules of PPINs, and provide a comparative study of these differential networks using two examples; one for HD and another for cancers. The general philosophy of this analysis is depicted schematically in Fig. 5. In this figure, the wild type protein interacts with many other proteins forming a complex interaction network. Broadly, the schematic wild type network has three subgraphs or modules (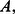 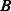 and 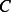); proteins in each module are marked there with identical colours. The mutant protein loses some proteins as interacting partners (marked as pink) and gains some new ones (marked as orange, blue and violet). The network of the mutated protein has a revised modular structure 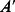, 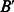 and 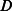. Module 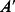 and 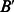 are re-structured and they have some proteins from other modules and some new proteins. Module 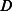 is gained by the mutation as most of proteins in this module were not present in the wild type network, and module 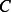 is lost. Correspondingly, the biological processes (GO terms) which are enriched in module 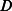 are gained and those enriched in module 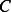 are lost. We argue that this loss or gain of BPs lead to loss or gain of functions in the pathogenesis of the mutation induced disease.

In this article we explained the general idea of 'obtaining the loss and gain of functions from the loss and gain of BPs enriched in protein modules' using two examples; one for HD and another for cancers. Our analysis predict a set of broadly classified biological processes (from the the GO terms enriched in the modules of HTT and TP53 networks) which could be involved in the pathogenesis of HD and cancers respectively. In HD, the broadly classified BPs, like post transcriptional regulation of genes, apoptosis, synaptic transmission, JNK pathway, transcription deregulation, glucose transport, histone modifications etc are enriched with the proteins in modules of wHTT and mHTT networks. These BPs are already known to be altered in HD pathogenesis. Similarly, the gain and loss of BPs mTP53 results in the metastatic properties, which have been observed recently.

Although, we demonstrated the plausible loss and gain of biological processes in two examples where mutation alters protein interaction networks of wild type protein, the methodology discussed here can be adopted and applied to study differential PPIN in general. In particular, knowing the changes in the protein interaction network, either due to mutations that modify the structure of the protein at the binding surface or due to the change in interaction environments, one can predict what alteration might occur in the biological processes and functions. Such analysis may help understanding the loss or gain of biological processes/functions in genetic diseases caused by mutations. This may in future lead to better design of disease intervention through targeting the biological processes/functions of specific modules.

## Supporting Information

Dataset S1 **Differential interaction of the wHTT and mHTT protein.**
(XLS)

Dataset S2 **The proteins belonging to different modules of wHTT network and their GO term enrichment analysis.**
(XLS)

Dataset S3 **The proteins belonging to different modules of mHTT network and their GO term enrichment analysis.**
(XLS)

Dataset S4 **The list of LOF,GOF and GOF/LOF for wHTT and mHTT networks.**
(XLS)

Dataset S5 **Differential interaction of the wTP53 and mTP53 protein.**
(XLS)

Dataset S6 **The proteins belonging to different modules of wTP53 network and their GO term enrichment analysis.**
(XLS)

Dataset S7 **The proteins belonging to different modules of mTP53 network and their GO term enrichment analysis.**
(XLS)

Dataset S8 **The list of LOF,GOF and GOF/LOF for wTP53 and mTP53 networks.**
(XLS)

Dataset S9 **The GeneDeck analysis of the proteins in the modules of wTP53 and mTP53 networks and enrichment of metastatsis.**
(XLS)

Text S1 Text 1, Differential interaction due to mutation in HTT and TP53. Text 2, Analysis of network structure. Text 3, Enriched biological processes in modules. Text 4, Enrichment of metastasis from GeneDeck.
(PDF)
