# Supplementary material for: Comparison of Modules of Wild Type and Mutant Huntingtin and TP53 Protein Interaction Networks: Implications in Biological Processes and Functions
Source: PLoS One. 2013 May 31;8(5):e64838. doi: 10.1371/journal.pone.0064838 (PMC3669416; doi:10.1371/journal.pone.0064838)
Supplement: Text S1 — Text 1, Differential interaction due to mutation in HTT and TP53. Text 2, Analysis of network structure. Text 3, Enriched biological processes in modules. Text 4, Enrichment of metastasis from GeneDeck. (PDF) [file pone.0064838.s010.pdf]

Supporting Information

Mahashweta Basu[1], Nitai P. Bhattacharyya[2], P. K. Mohanty[1]
1 Theoretical Condensed Matter Physics Division,
Saha Institute of Nuclear Physics, 1/AF Bidhan Nagar, Kolkata 700064, India
2 Crystallography and Molecular Biology Division,
Saha Institute of Nuclear Physics, 1/AF Bidhan Nagar, Kolkata 700064, India

Text 1 : Differential interaction due to mutation of HTT and TP53

Huntingtin protein

The mutated huntingtin protein mHTT preferentially interact with a set of different proteins compared to the wild type HTT (wHTT). The data for this differential interactions are curated from literature as follows. Primarily we have collected 328 HTT-interacting proteins identified by yeast 2 hybrid (Y2H) assays [1–3], affinity pull down followed by mass spectrometry assay [3] and proteins in the aggregates of mutant HTT [4]. Since Y2H assay results in false positives [5], we consider only 154 HTT interacting proteins that are validated by (i) second method like co-immunoprecipitation or (ii) the interacting proteins are tested for their implications in HD pathogenesis in cell or animal models of HD. Out of these sorted 154 HTT interacting proteins 17 proteins are seen to interact with wHTT, 12 proteins are identified in the aggregates of mutant HTT and 25 proteins interact preferentially with mutant HTT. Thus in totality, wHTT interact with 17 proteins whereas mHTT interacts with completely new 37 proteins. The list of these protein along with other subsidiary informations are presented in Dataset S1 (sheet 1).

The PPIN of wHTT is constructed by taking 306 proteins (wHTT, its 17 primary and 288 secondary interactors) and interactions among them, whereas the PPIN of mHTT has 1542 proteins (see Dataset S1 (sheet 2 and sheet 3)). Corresponding networks, drawn using Cytoscape [6], are shown in Fig. S1.

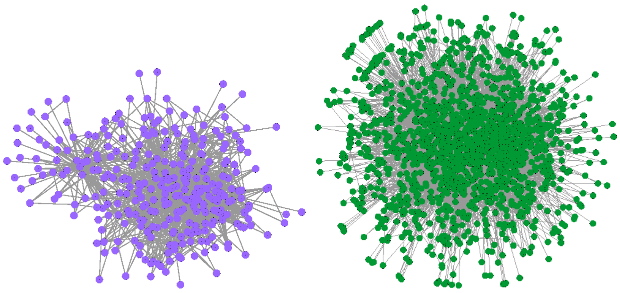

Figure S1. The interactome of wHTT and mHTT involves 306 and 1542 proteins respectively. If a pair of proteins present in a particular set has interaction between them, then a link is constructed between these two proteins(nodes). Exhausting every pair of proteins for both the sets we end up in constructing the interactome of the wHTT (left) and mHTT (right) using Cytoscape software [6].

Common proteins in wHTT and mHTT networks

Out of the 17 primary interactors of wHTT, 8 proteins namely (DLG4, CASP3, CTBP1, EIF2C2, REST, ZDHHC17, MEF2D, HIP1) change their direct interaction with wHTT and get involved in mHTT PPIN as secondary interactors. Whereas out of 288 secondary interactors in wHTT PPIN, 10 proteins now

directly interact with mHTT. The list of these proteins are (PARK2, CASP10, TP53, CREBBP, SIN3A, CASP2, NFYC, CASP8, SP1, TBP). Also it is to be noted that, there are 107 proteins remain as secondary interactors in both the wHTT and mHTT PPIN (list given in Table S1).

TP53 protein

TP53 is a tumor suppressor gene. The mutation of this gene causes cancer. In a recent work Coffill *et. al.* [7] have studied the biological effects resulting from the missense mutations in TP53 (p53R273H). Their experimental results indicate that wTP53 and mTP53 differentially interact with 17 and 30 proteins; list of these proteins are given Dataset S5 (sheet 1).

The PPIN of wTP53 is constructed by taking 619 proteins (wTP53, its 17 primary and 601 secondary interactors) and interactions among them, whereas the PPIN of mTP53 has 578 proteins (see Dataset S5 (sheet 2 and sheet 3)). The corresponding networks, drawn using Cytoscape [6], are shown in Fig. S2.

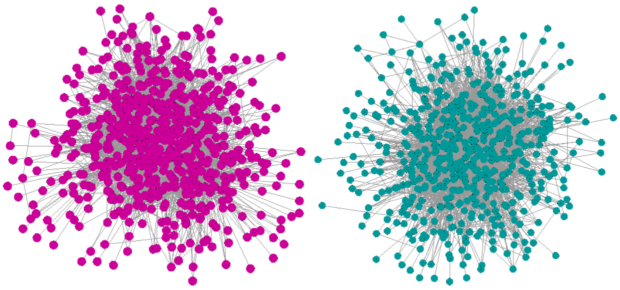

Figure S2. The PPIN of wTP53 and mTP53 interacting proteins are constructed using the Cytoscape software [6]. The network in the left is for the wild TP53 and in right is for mutant TP53.

Common proteins in wTP53 and mTP53 networks

Similar to the previous case the mutation of TP53 causes change in interacting partners of TP53, altogether resulting in different secondary interactors. Wild TP53 primarily interact with 17 proteins, among them 5 proteins (YWHAG, BAG6, TUBB3, RPL5, MDM2) lose their interaction with wTP53 and get involved in mTP53 PPIN as secondary interactor. Again these 7 proteins (PARD3, PLK1, KHDRBS1, CSNK2A1, PSMD2, CSNK2B, PSMC1) which were secondary interactors in wTP53 PPIN now interact with mTP53 directly. Apart from these there are 111 proteins common to both wild and mutant TP53 network, and are secondary interactors for both the networks (listed in Table S1).

Text 2 : Analysis of network structure

In recent years theory of complex networks has been used enormously in various fields [8]. The social systems (friendship, mobile or citation networks), information systems (World Wide Web and router networks), biological systems (neural, protein folding and gene regulatory networks) are a few to mention. Network theory provides a quantifiable description for comparison and characterization of complex networks. Here we shall focus on some robust measures of network topology : the degree distribution, the clustering coefficient, the average path length and the diameter.

Degree Distribution: The most elementary characteristic of a node (vertex) is its degree. The degree of a vertex k in a network is defined as the number of edges connected to that vertex. The degree distribution, $P(k)$, gives the probability that a selected node has exactly k links. $P(k)$ is obtained by

counting the number of nodes with $k = 1, 2, \ldots$ links and then normalizing by the number of nodes N. The degree distribution of many real world networks, including PPINs [9] are commonly scale free, *i.e.*

$$P(k) \sim k^{-\gamma}.$$

The degree distribution $P(k)$ for all four PPINs, *i.e.* wild and mutant networks of HTT and TP53, are found to be scale free (see Fig. S3), with the exponent $\gamma \simeq 2$. The average degree and our best estimate of the exponent γ are listed in Table S2.

Clustering coefficient: In a highly dense network, the neighbours of a given node are very likely to be connected among themselves. The clustering coefficient

$$c_i = \frac{2E_i}{k_i(k_i - 1)}$$

gives a measure of how connected are the neighbours of node i. Here E_i the actual number of links which connects all the k_i neighbours of node i and $k_i(k_i - 1)/2$ is the maximum number of possible links. Clearly the maximum value of c_i is unity. The average clustering coefficient of a network of N nodes is

$$C = \frac{1}{N} \sum_{i}^{N} c_i,$$

which gives a notion of how dense is the network, with maximum value 1 corresponding to a clique (*i.e.* all N nodes are connected to each other).

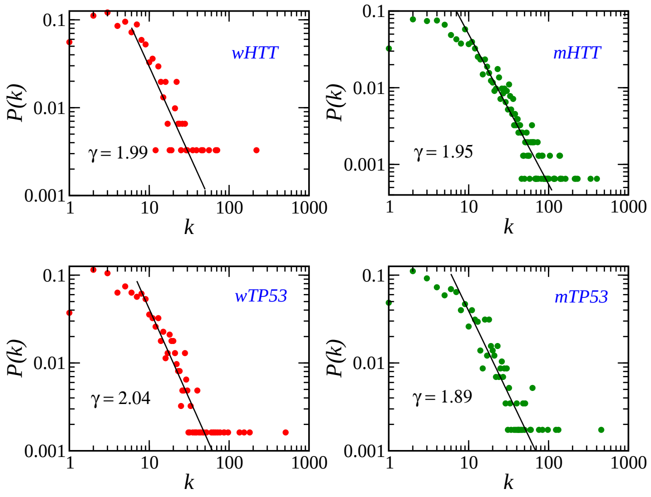

Figure S3. Degree distribution of wild and mutant HTT and TP53 network. All the four network follows scale free degree distribution. The corresponding power-law exponent is written in the graph itself.

Average path length : In a connected network, a given node i can be reached from any other node j by several alternative paths. The shortest path $l(i, j)$ is the path with the smallest number of links between nodes i and j. The average path length of a network of N nodes, thus $N(N - 1)/2$ vertex pairs, is

$$L = \frac{\sum_{i \neq i}^{N} 2l(i, j)}{N(N - 1)}.$$

The network diameter : The network diameter D is the largest of the shortest paths among all the vertex pairs. In other words, D is the length of the 'longest' shortest path in the network. The average path length and diameter for the networks of wHTT , mHTT, wTP53 and mTP53 are calculated and their values are listed in the Table S2.

Modular structure of network : Other than these network parameters, detection of modules or community structures is widely used to reveal the organizational principles of nodes within the context of the entire system. Modules are specific groups of nodes which has larger density of links within the group than outside it. Each of such modules can be regarded as a discrete entity whose function or properties are in some way separable from other modules. A large variety of community detection techniques [10] have been developed based on centrality measures, link density, percolation theory etc.

The most obvious way of finding groups in a network is to minimize the number of edges connecting the groups. Recently, Newman *et.al.* [11] proposed a method of finding community structure of a network by associating a score called modularity Q for each possible partition of a network; a good partition of a network can be obtained by maximizing Q. It is argued that simply rearranging the network, such that only few edges exist between the communities, is not enough. Rather one must rearrange it in a way that the communities are connected with fewer than expected edges. Based on this, Newman *et. al.* defined a score called modularity Q which is proportional to the number of edges present within the groups minus the expected number in an equivalent random network.

To be more precise, let us consider an undirected network of N nodes labeled by $i = 1, \dots N$, having an adjacency matrix A with boolean elements,

$$A_{ij} = \begin{cases} 1, & \text{if nodes } i \text{ and } j \text{ are connected} \\ 0, & \text{otherwise.} \end{cases}$$

Thus, the degree of the node i is $k_i = \sum_j A_{ij}$, and the total number of links $m = \frac{1}{2} \sum_i k_i = \frac{1}{2} \sum_{ij} A_{i,j}$. If the network is to be partitioned into groups, the modularity is defined by

$$Q = \frac{1}{2m} \sum_{ij} \left(A_{ij} - \frac{k_i k_j}{2m} \right) \kappa_{i,j}, \tag{1}$$

where $\kappa_{i,j} = 1$ (or 0) if nodes i and j belong to same (different) partitions. Note that $k_i k_j / 2m$ is the expected number of links between nodes i and j, if edges were placed at random and it assures that Q is maximum when two groups are connected by smaller than expected number of links. A positive values of Q indicates the possible presence of community structure(s) and one need to look for a partition for which the modularity is preferably large and positive. In the following we apply this procedure to obtain modular structure of wHTT, mHTT, wTP53 and mTP53 networks.

Modularizing the wHTT and mHTT network we see that wHTT network contains 7 and mHTT network contains 8 modules. Also the wTP53 and mTP53 networks divides into 4 and 5 modules. The size of each modules *i.e.* number proteins present in each modules are given in the Table S2. The names of the proteins that constitute the modules of wild and mutant HTT network are provided in the Dataset S2 (sheet 1) and S3 (sheet 1) respectively. Similarly for TP53, the name of the proteins present in the modules of the wild and mutant TP53 networks are given in the Dataset S6 (sheet 1) and S7 (sheet 1) respectively.

Text 3 : Enriched biological processes in modules

To find the possible function(s) of the modules, we utilized bioinformatics enrichment tools. There are many tools available for such enrichment analysis, we mainly used GeneCodis3 [12] for the convenience.

GeneCodis is a web-based tool for interpretation of large scale data integrating from different sources like Gene Ontology (GO, pathways in Kyoto Encyclopedia of Genes and Genomes (KEGG), PANTHER

pathways, Online Mendelian inheritance in Man and others). Given a set of genes in the query field, GeneCodis associates GO terms for each gene and calculate whether the fraction of genes in a particular GO term among the input gene list is over represented compared to the background frequency (the total number of genes involved in that GO term over the total number gene in the organism). It further corrects the significance level for multiple testing. When the list of proteins present in a given module is feed to GeneCodis3 as the query set, it gives a list of associated biological processes (GO terms) and their respective significance values. In this study we considered only those GO terms which have $p < 0.0001$.

HTT PPIN : The set of proteins present in each of the 7 and 8 modules of wHTT and mHTT PPINs are analyzed with GeneCodis3. The GO terms associated with each of the modules are enlisted in Dataset S2 and S3 according to high to low significance. The GO terms with corrected significance value greater than 0.0001 are discarded. Thus the number of GO terms belonging to each of the modules of wHTT PPIN are $W1(7)$, $W2(24)$, $W3(47)$, $W4(7)$, $W5(32)$, $W6(13)$, $W7(3)$ and for mHTT PPIN are $M1(161)$, $M3(78)$, $M5(198)$, $M6(12)$, $M7(5)$. For the modules $M2$, $M4$ and $M8$, all the GO terms that are obtained after GeneCodis analysis have p-value greater than 0.0001, so these GO terms are not considered for further analysis. Accumulating all the GO terms involved in the modules of wHTT and mHTT PPIN, one gets 129 and 389 GO terms respectively. (Note that merely adding the number of GO terms for each modules separately for wHTT and mHTT networks gives numbers greater than that mentioned; reason for this is that a single GO term can occur simultaneously in two or more modules of wHTT or mHTT network.) A detailed study reveals that both these set of GO terms obtained for wHTT and mHTT modules has 65 GO terms in common. Thus one can conclude that the unique GO terms that are involved only in wHTT and mHTT network are $64(= 129 - 65)$ and $324(= 389 - 65)$. The Biological processes relating to these 64 GO terms in wHTT network are lost due to mutation-LOF, whereas the BPs corresponding to 324 GO terms are GOF, and BPs for the common ones 65 are GOF/LOF both. We group the similar GO terms under a single broad BP. A clear data demonstrating these LOF, GOF and GOF/LOF (see Table S3) is given Dataset S4.

TP53 PPIN : A similar study for the module of wild and mutant TP53 PPIN modules is being done to obtain the LOF, GOF and GOF/LOF separately. The total number of GO terms (considering GO terms with $p < 0.0001$ for all the modules of wTP53 and mTP53 separately) that are present for wild and mutant TP53 networks are 127 and 172 respectively. These two sets has 70 GO terms in common. Thus the unique GO terms present for the wild and mutant TP53 are 57 and 102; these are responsible for LOF and GOF function respectively. For the details of the loss and gain of functions refer to Table S4 and Dataset S8.

Text 4 : Enrichment of metastasis from GeneDeck

Metastasis, which is shown as GOF in mutation of TP53 [7], is not described for "biological process" in Gene Ontology term. We have used another tool, GeneDecks [13], which provides a similarity metric by highlighting shared descriptors between genes, based on annotation within the GeneCards compendium of human genes. Given a set of genes as query to the field Set Distiller in GeneDecks (Version 3), an online analysis tool that provides output of various descriptors like various cancers and diseases with the attribute Disorder taking data from different resources like Alma Knowledge Server, PharmGKB, The Breast Cancer Gene Database, Tumor Gene Database, GeneTests (formerly GeneClinics), OMIM, Swiss-Prot and Genatlas. The proteins present in the modules of wTP53 and mTP53 are analyzed using GeneDeck in order to get the information about the presence of metastasis in the corresponding modules. It is seen that the almost all the modules of wTP53 (except module $W4$) and mTP53 shows enriched metastasis (refer to Dataset S9).

References

1. Faber PW, Barnes GT, Srinidhi J, Chen J, Gusella JF, et. al. (1998) Huntingtin interacts with a family of WW domain proteins. Hum Mol Genet 7:1463-1474.

2. Goehler H, Lalowski M, Stelzl U, Waelter S, Stroedicke M, et. al. (2004) A protein inter- action network links GIT1, an enhancer of huntingtin aggregation, to Huntingtons disease. Mol Cell 15:853-65.

3. Kaltenbach LS, Romero E, Becklin RR, Chettier R, Bell R, et. al. (2007) Huntingtin interacting proteins are genetic modifiers of neurodegeneration. PLoS Genet 3, e82.

4. Mitsui K, Nakayama H, Akagi T, Nekooki M, Ohtawa K, et. al. (2002) Purification of polyglutamine aggregates and identification of elongation factor-1alpha and heat shock protein 84 as aggregate-interacting proteins. J Neurosci 22:9267-9277.

5. Von Mering C, Krause R, Snel B, Cornell M, Oliver SG, et. al. (2002) Comparative assessment of large-scale data sets of protein-protein interactions. Nature 417:399-403.

6. Shannon P, Markiel A, Ozier O, Baliga NS, Wang JT, et. al. (2003) Cytoscape: a software environment for integrated models of biomolecular interaction networks. Genome Res 13:2498-2504.

7. Coffill CR, Muller PA, Oh HK, Neo SP, Hogue KA, et. al. (2012) Mutant p53 interactome identifies nardilysin as a p53R273H-specific binding partner that promotes invasion. EMBO Rep 13:638-644.

8. R. Albert, A. Barabási (2002) A Statistical mechanics of complex networks. Rev of Mod Phys 74:47-97.

9. R. Albert (2005) Scale-free networks in cell biology. J Cell Sci. 118:4947-4957.

10. Fortunato S (2010) Community detection in graphs. Phys Rep 486:75-174.

11. Newman MEJ, Girvan M (2004) Finding and evaluating community structure in networks. Phys Rev E 69, 026113.

12. Huang Da W, Sherman BT, Lempicki RA (2009) Bioinformatics enrichment tools: paths toward the comprehensive functional analysis of large gene lists. Nucleic Acids Res. 37:1-13.

13. Safran M, Dalah I, Alexander J, Rosen N, Iny Stein T, et. al. (2010) GeneCards Version 3: the human gene integrator, Database (Oxford). 2010 Aug 5;2010:baq020.

Table S1. List of the secondary interacting proteins that are common in to both wild and mutant PPIN of HTT and TP53 separately.

Common	Protein name
wHTT-mHTT(107)	YWHAQ, NFATC2, BCL3, HIST3H3, SMARCA4, TBK1, MAPK1, BTRC, SMARCC2, APC, PRKCA, CTTN, SMARCE1, UBC, MEN1, WAS, TCF7L2, KDM1A, NEUROD1, CASP6, RBBP5, GTF2E2, CBL, ZEB1, NUP98, AKT1, NUB1, IFT57, DHX58, BLM, TUBG1, EHMT1, COPS5, EHMT2, KDM5C, MAPK14, PSMC3, CFLAR, CARM1, NCOR1, CASK, MDM2, PRMT5, NCOA3, HSPB1, CDC42, BRCA1, HTR2C, HSPD1, FZD7, TBL1X, UBE2I, SENP3, HDAC1, HDAC2, HEY2, HDAC3, HDAC4, SUB1, SREBF2, HDAC5, TP73, THAP11, EP300, BID, HDAC9, TCF4, GTF2B, WIPF1, EGFR, KAT2A, SNCA, KAT2B, MLL, PFDN1, GRIK5, SERPINH1, RPS10, DNM1, SUMO1, SUMO2, USO1, KAT8, CHD3, RAC1, SIN3B, IKZF1, HIC1, DBNL, GRIN2B, IKZF4, SOX2, EIF6, YWHAE, DICER1, MAP3K14, BIRC2, MECOM, TGIF1, KHDRBS1, BIRC5, CABIN1, SATB1, HGS, ABL1, BCL2, XIAP
wTP53-mTP53(111)	UBC, TANK, RBBP4, RBBP7, PPP4C, MAGED1, SETDB1, SMAD1, UIMC1, CDH1, TERF1, NOLC1, PSMD10, CCDC85B, UBE2I, USP42, IKBKB, WRAP73, DCAF7, E2F1, MGMT, NCL, HSP90AA1, PRMT1, SRRM1, SRRM2, CHEK2, FBXW11, ITCH, PIK3R1, RB1, KIF5C, YWHAZ, STUB1, ARRB1, EP300, AR, SNAI1, RPS6KB1, MAP3K5, ESR1, UCHL1, UCHL5, STAU1, PCK1, MDM4, RPA1, RPA2, RPAP1, BCL3, CDC5L, RASSF8, HSPA1A, PSMA3, NCOR2, PSMA7, TP53BP1, NDRG1, EXO1, CASP8, ARAF, H2AFX, PRPF40A, PELP1, EIF2C3, PIN1, NR3C1, CLSPN, RAD21, NEDD4, NEDD8, IMMT, CHAF1A, TSC1, MTA1, PUF60, CTBP1, MLLT4, MAPK6, TNFRSF1A, MEPCE, ARR3, SRGAP2, CREBBP, SRC, PRKCZ, SFN, CDK9, ATM, WNK1, RIPK1, SUMO1, SUMO2, HDGF, BTRC, LRIF1, CDKN1A, MDC1, RNPS1, PPP2R2D, HIST1H3A, HDAC1, HDAC3, DISC1, HDAC5, HDAC6, TP73, TAF1, PSMC4, PSMC5, C5ORF25

Table S2. Comparison of network properties between wild-type and mutant PPIN of HTT and TP53.

network	node	edge [1]	$\langle k \rangle$	γ	C	d	L	No. of Modules: Module sizes	Q
wHTT	306	1380+17	9.13	1.95	0.436	4	2.418	7: 18, 66, 79, 18, 82, 8, 35	0.415
mHTT	1542	13105+37	17.05	1.99	0.361	4	2.349	8: 643, 3, 377, 2, 485, 7, 22, 3	0.302
wTP53	619	3718+17	12.07	2.04	0.452	4	2.232	4: 204, 151, 183, 81	0.331
mTP53	578	3521+30	12.29	1.89	0.406	4	2.282	5: 193, 127, 25, 111, 122	0.338

[1] The total number of edges is written as the sum of the secondary links and primary links.

Table S3. LOF, GOF, LOF/GOF occurred due to mutation of HTT.

GO terms related to	Modules of mHTT network (unique GO terms)	Modules of wHTT network (unique GO terms)	Modules of wHTT-mHTT network (unique GO terms)
Cell cycle	M1(4),M3(12),M5(5)	W2(1),W6(3)	(W2,W5)-(M1,M3,M5)(4)
Signaling	M1(30),M5(31),M6(2)	W3(17),W4(4)	(W3,W4,W5,W7)-(M1,M5)(9)
Transcription processes and regulation	M1(5), M3(5), M5(31)	W7(1)	(W1,W5,W7)-(M3,M5,M6(17)
Apoptosis	M1(11)	W2(1)	(W2,W5)-(M1,M3,M5)(12)
DNA replication	M3(12)	-	-
DNA damage and repair	M1(6),M3(17),M5(4)	-	(W3,W5)-(M1,M3,M5)(4)
Immunological	M1(7)	-	-
Protein folding	M1(7)	-	-
Autophagy	M1(5)	-	-
Translation	M1(3)	-	-
Metabolism	M1(1)	-	-
Development and differentiation	M1(4),M5(57),M6(8)	-	(W3,W5)-M5(5)
Cell migration and shape	M1(4)	-	-
Proteasomal degradation	M1(14)	W2(1)	(W2,W5)-(M1,M3)(3)
Carbohydrate/Glucose transport/metabolism	M5(4)	W6(5)	-
Protein complex/membrane assembly/stabilization	M1(9)	-	-
Cell growth	M5(7)	-	-
Transcription from RNA polymerase III	M7(4)	-	-
Others	M1(4),M3(5),M5(6)	W3(4)	(W2,W3,W4,W5,W6)-(M1,M5)(6)
Gene silencing/micro RNA and RNA processing /translation	-	W1(5)	-
Synaptic transmission and neuronal activity	-	W3(12)	W3-M1(2)
Transport (ion/sugar)	-	W3(5)	-
Circadian rhythm	-	W2(1)	-
Protein /transmembrane transport	-	W6(4)	-
NFKB1 regulation	-	-	W2-M1(2)
Proliferation/growth	-	-	W5-(M1,M3,M5)(1)

Table S4. LOF, GOF, LOF/GOF occurred due to mutation of TP53.

GO terms related to	Modules of mTP53 network (unique GO terms)	Modules of wTP53 network (unique GO terms)	Modules of wTP53-mTP53 network (unique GO terms)
Cell cycle	M1(3),M2(4),M5(1)	W3(3)	(W1,W3,W4)-(M1,M2,M5) (14)
Signaling	M1(13), M5(7), M2(1)	W1(8), W3(1), W2(4)	(W1,W2,W3)-(M1,M4,M5) (9)
Transcription processes and regulation	M5(6)	W3(13), W4(2), W7(1)	(W1,W2,W3,W4)-(M1,M2,M5) (15)
Apoptosis	M5(1)	W2(2)	(W1,W2,W3,W4)-(M1,M2,M5) (4)
DNA replication	M2(11)	W3(3)	W3-(M2,M5) (3)
DNA damage and repair	M1(1), M2(14), M5(2)	W3(1)	(W1,W2,W3,W4)-(M1,M2,M5) (10)
Immunological	M2(3)	W2(3)	(W2,W4)-(M1.M2) (2)
Development and differentiation	M5(11)	-	W3-M5 (1)
Proteasomal degradation	M1(2),M2(3)	W1(1), W4(2)	(W4)-(M1.M2) (3)
Cell-cell communication	M1(5)	-	-
Cell proliferation/growth	M5(4)	-	W3-M5 (2)
Protein complex/membrane assembly/stabilization	M1(4)	-	-
Others	M1(2), M5(4)	W1(1), W4(4)	(W1,W2,W3,W4)-(M1,M2,M5) (5)
Translation	-	W1(1), W4(5)	-
Cell Migration and movement	-	W1(2)	-
Circadian rhythm	-	W4(1)	-
Metabolism	-	-	W4-M2 (2)
